# Supplementary material for: Unveiling IL6R and MYC as Targeting Biomarkers in Imatinib-Resistant Chronic Myeloid Leukemia through Advanced Non-Invasive Apoptosis Detection Sensor Version 2 Detection
Source: Cells. 2024 Apr 2;13(7):616. doi: 10.3390/cells13070616 (PMC11011921; doi:10.3390/cells13070616)

Supplementary information

Figure 1C

GFP

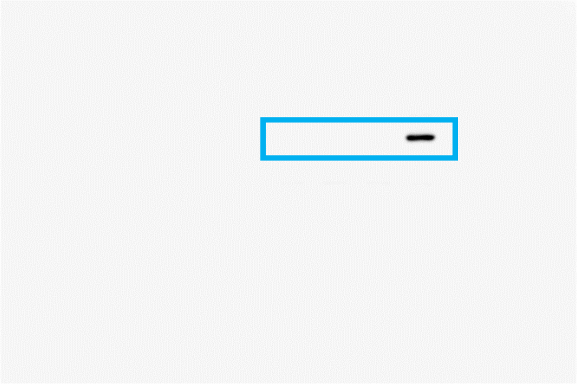

luciferase

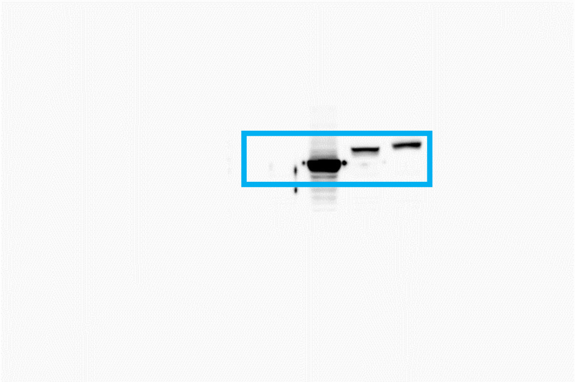

GAPDH

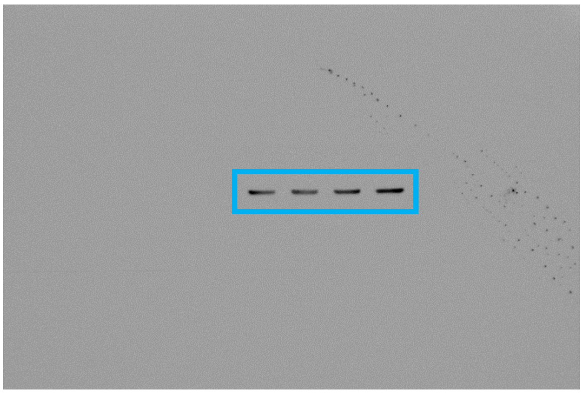

Figure 1F

p-ABL

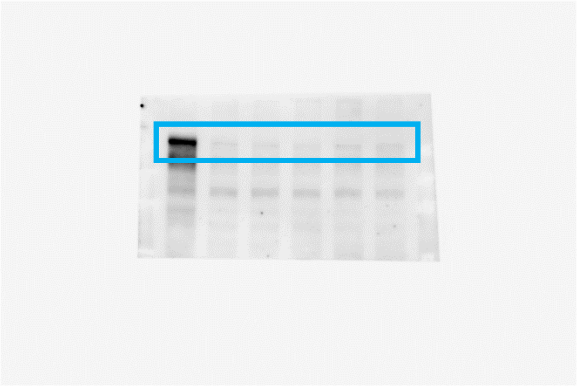

T-ABL

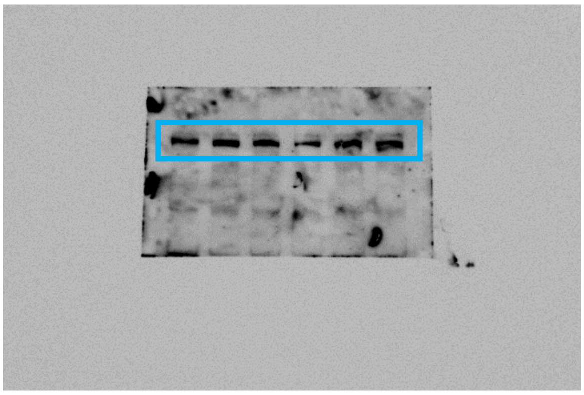

NIADS

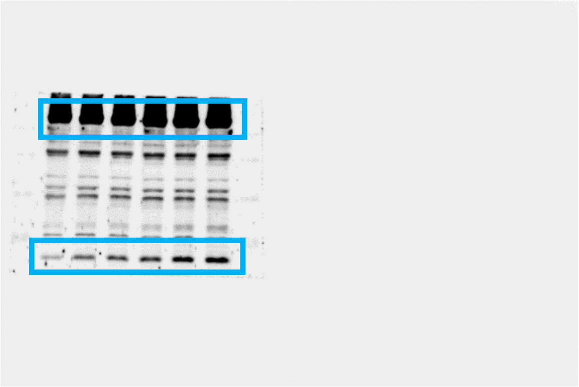

c-PARP

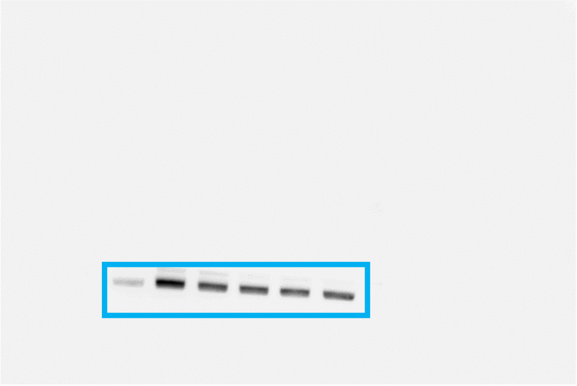

c-caspase 3

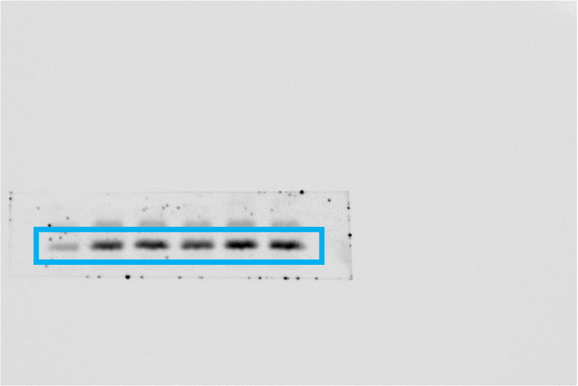

P21

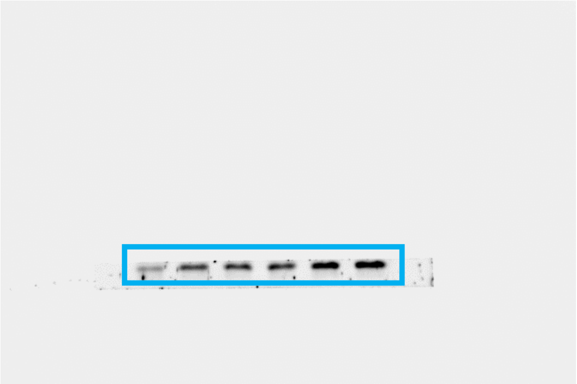

P27

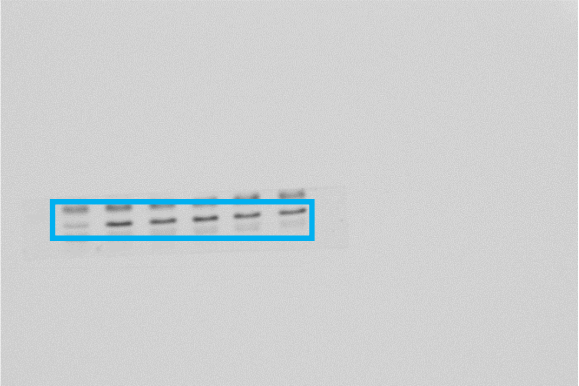

GAPDH

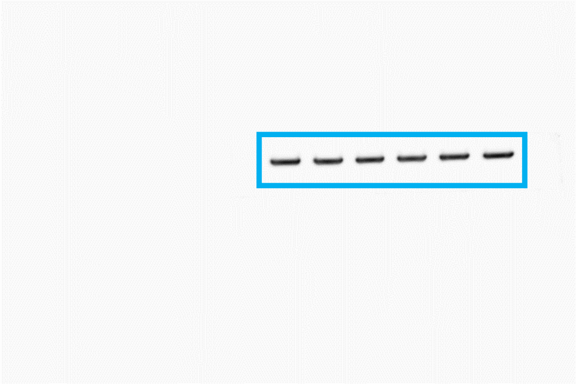

Figure 1G

p-ABL

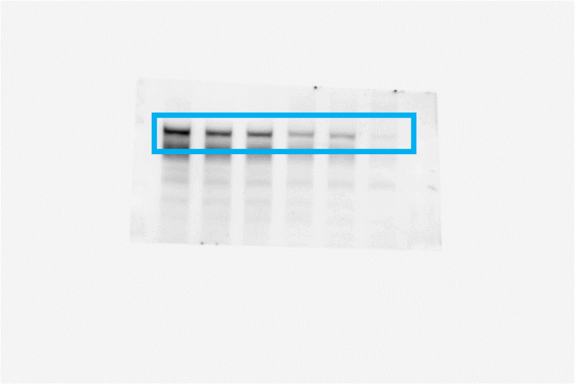

T-ABL

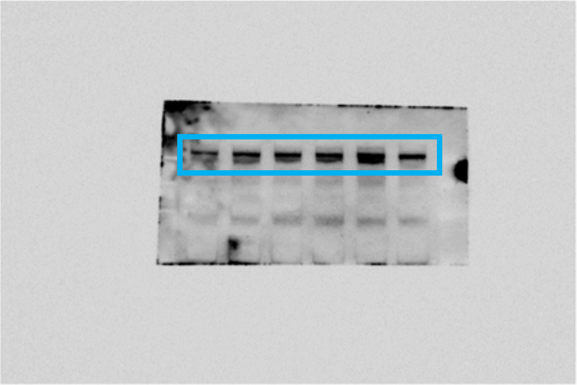

NIADS

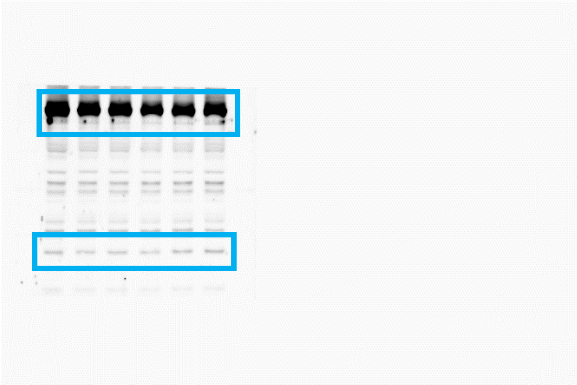

c-PARP

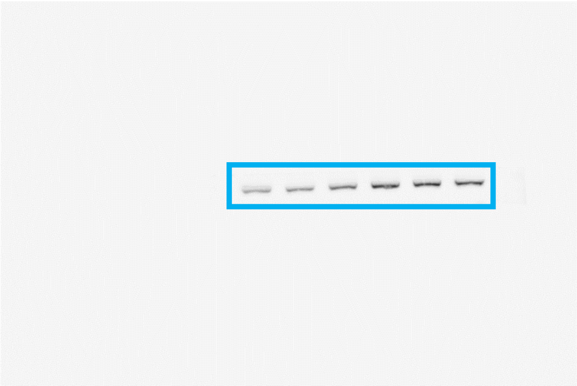

c-caspase 3

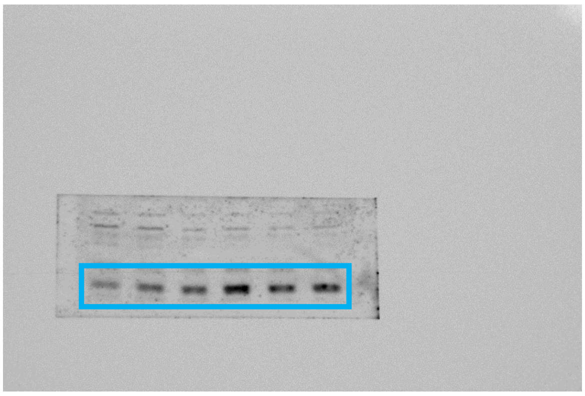

P21

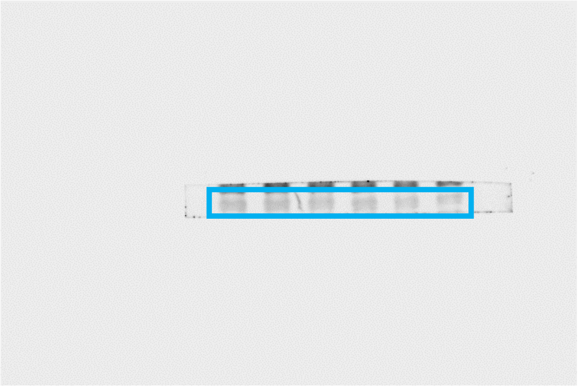

P27

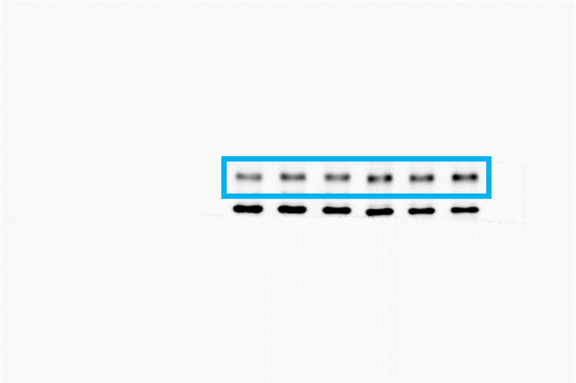

GAPDH

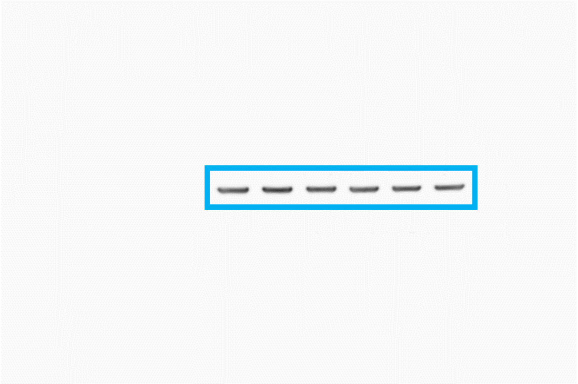

Figure 2D

p-ABL

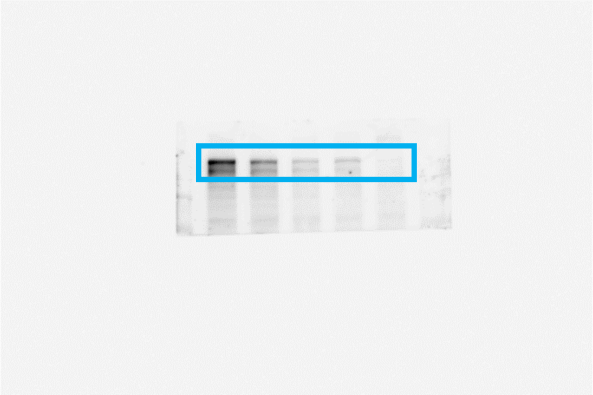

T-ABL

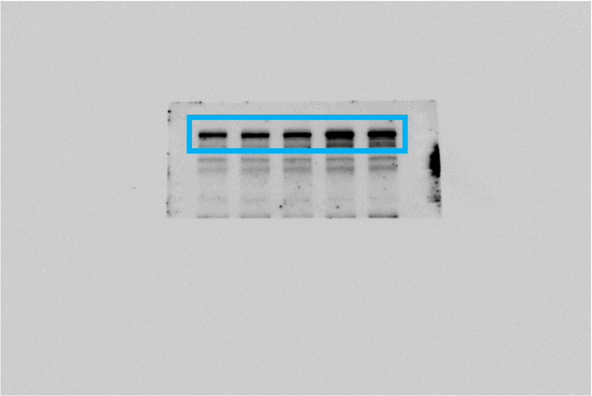

c.PARP

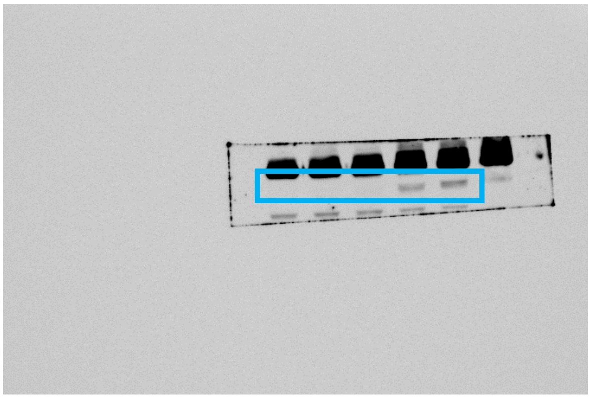

c.Caspase-3

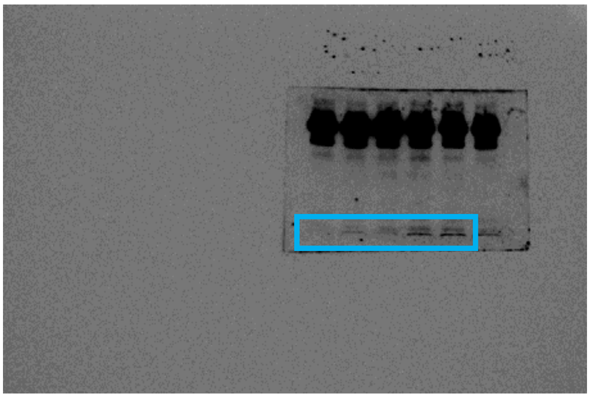

GAPDH

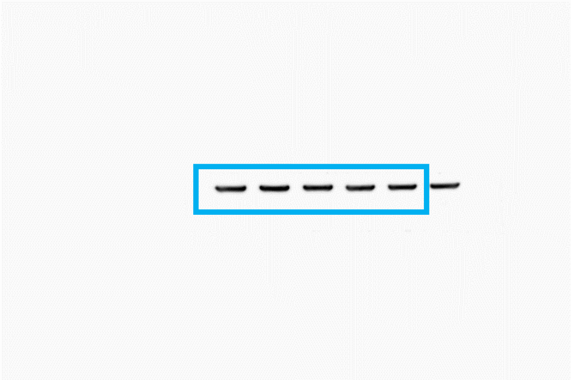

Figure 2E

p-ABL

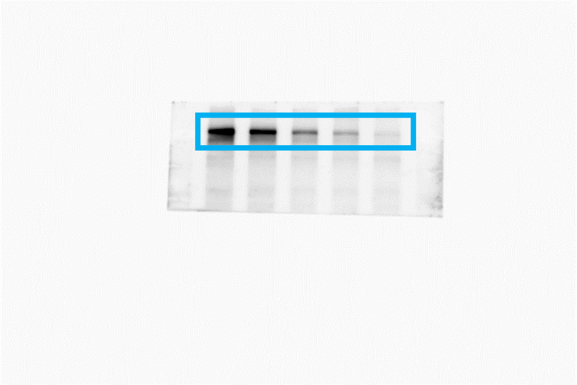

T-ABL

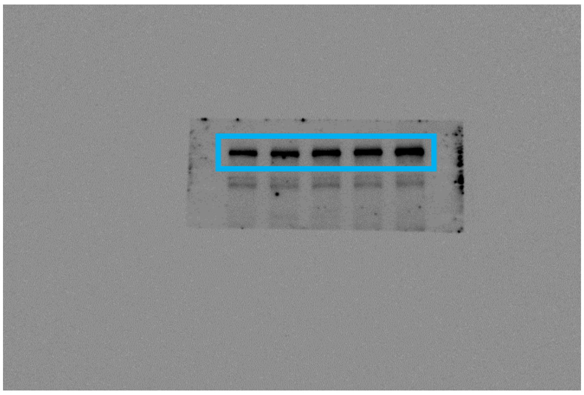

c.PARP

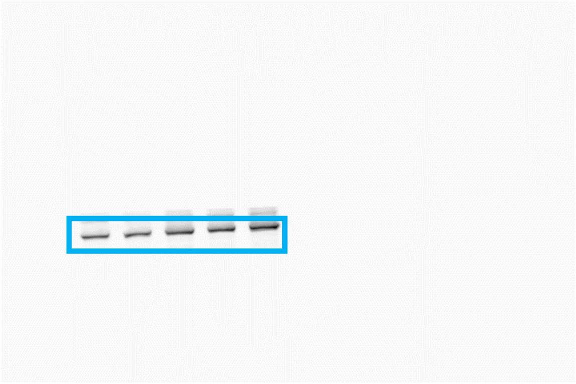

c.Caspase-3

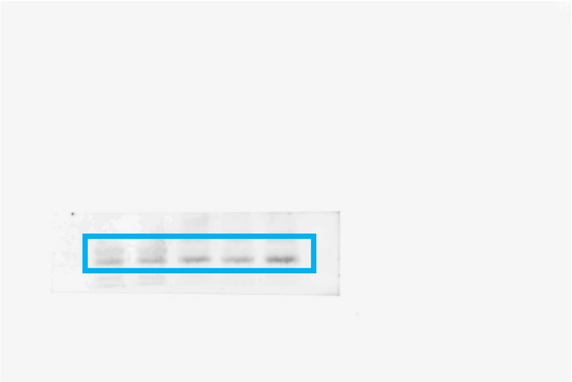

GAPDH

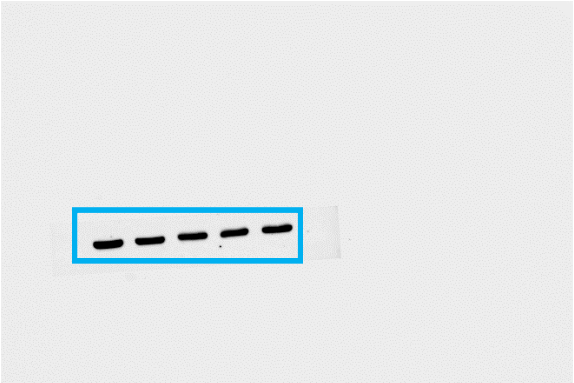

Figure 2F

p-ABL

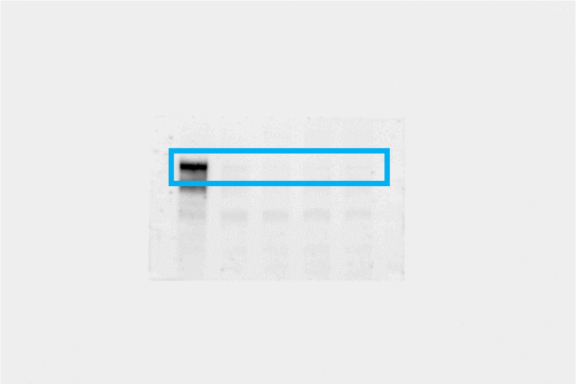

T-ABL

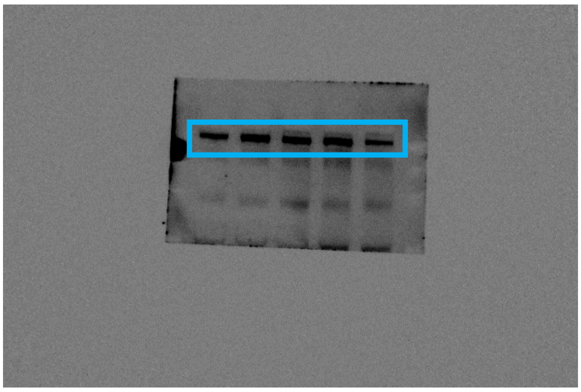

c.PARP

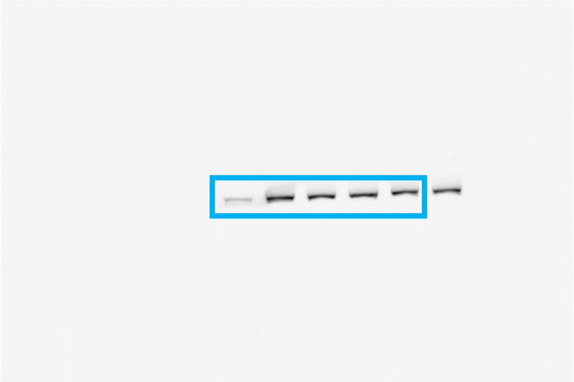

c.Caspase-3

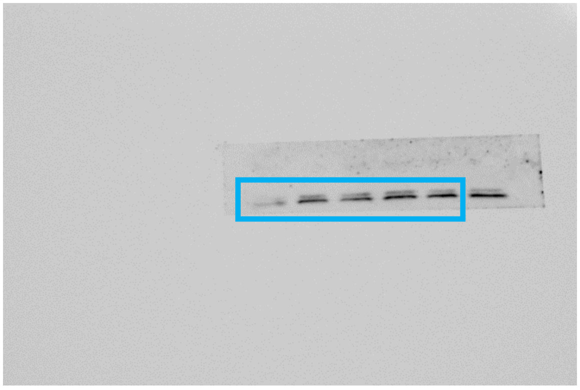

GAPDH

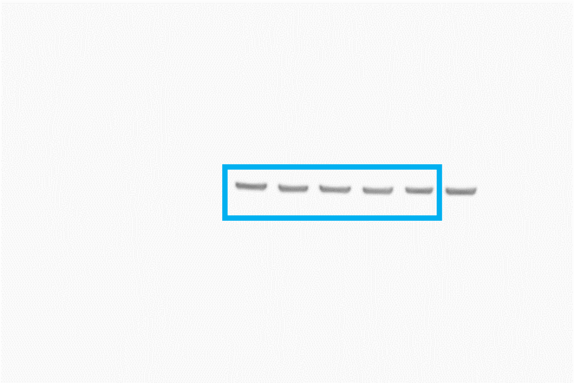

Figure 2G

p-ABL

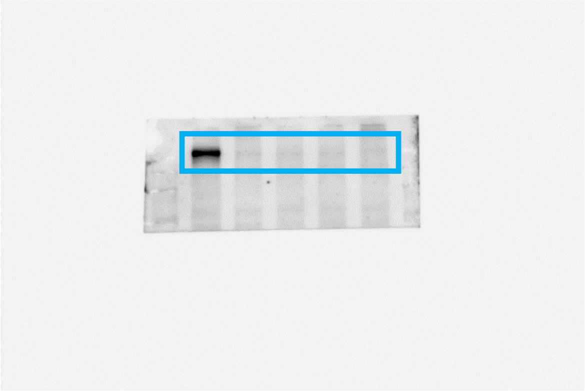

T-ABL

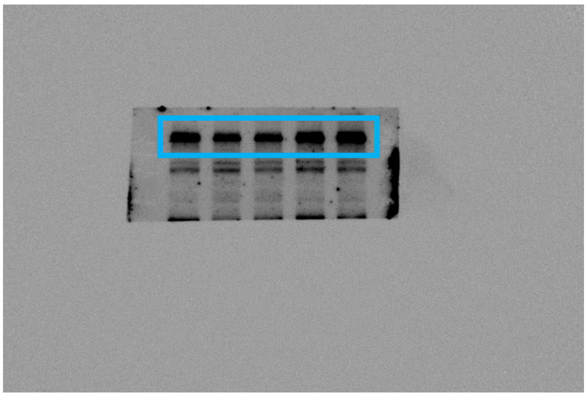

c.PARP

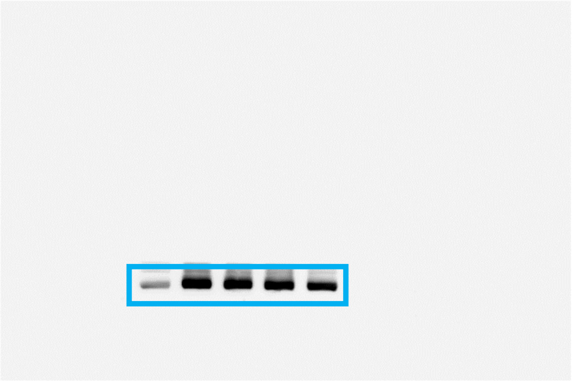

c.Caspase-3

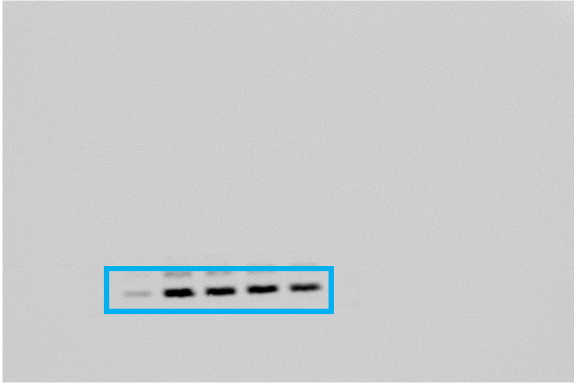

GAPDH

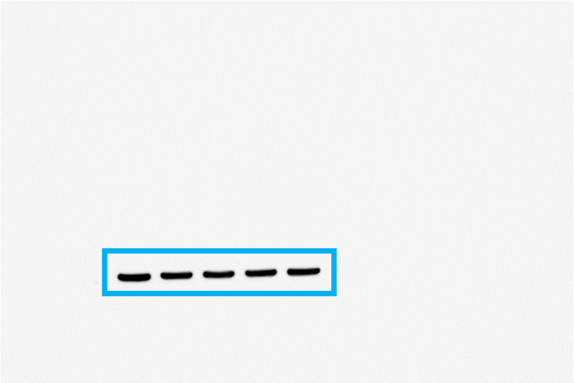

Figure 2J

IL6R

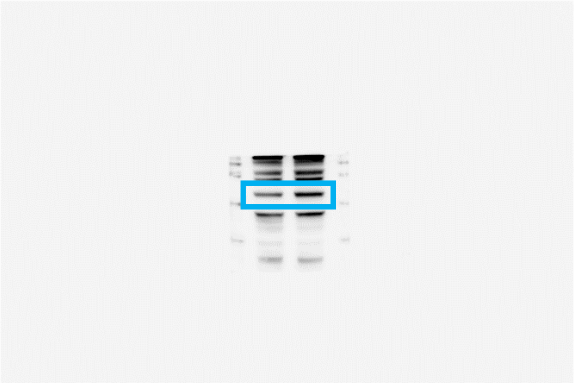

GAPDH

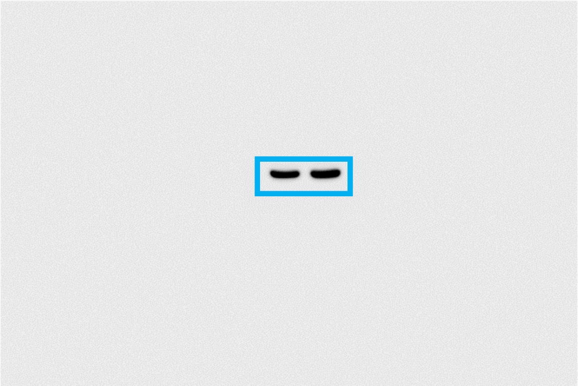

IL7R

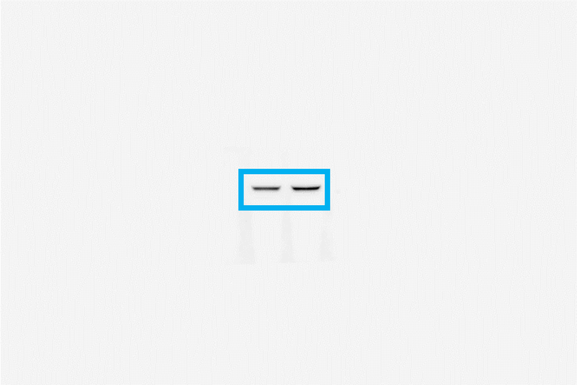

GAPDH

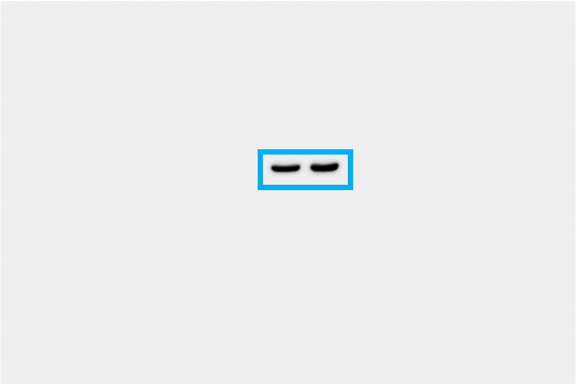

MYC

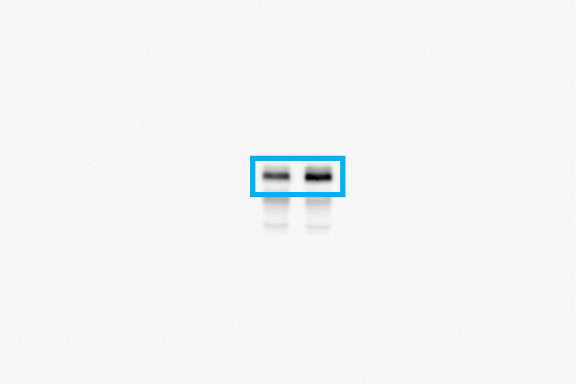

GAPDH

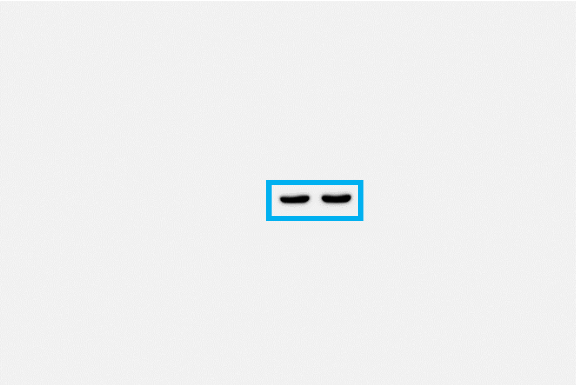

Figure 4B

IL6R

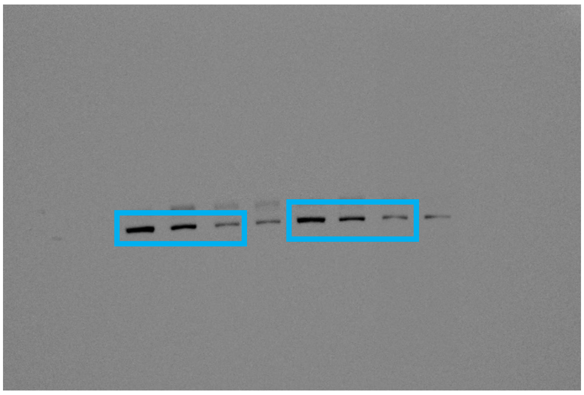

c.PARP

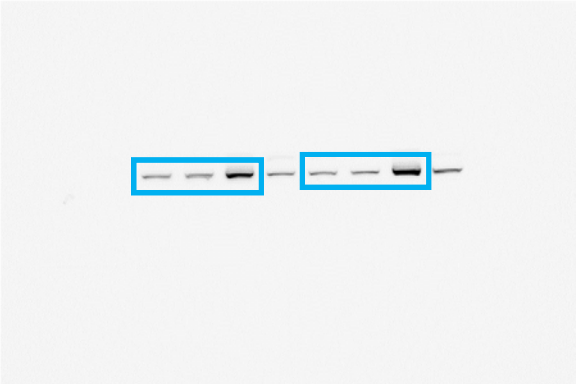

c.Caspase-3

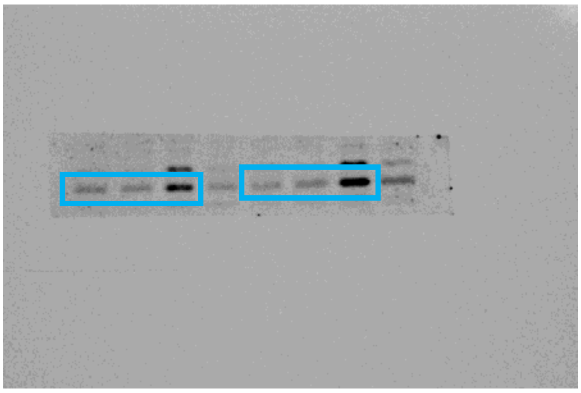

GAPDH

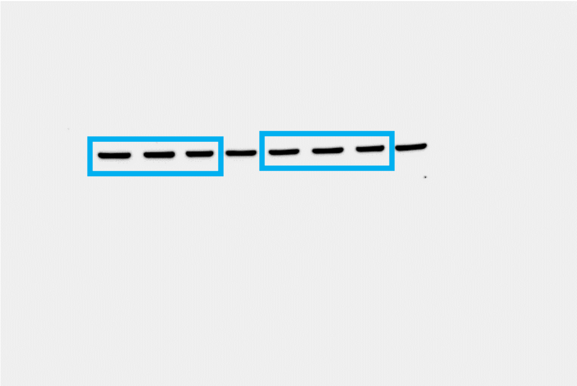

Figure 4C

IL7R

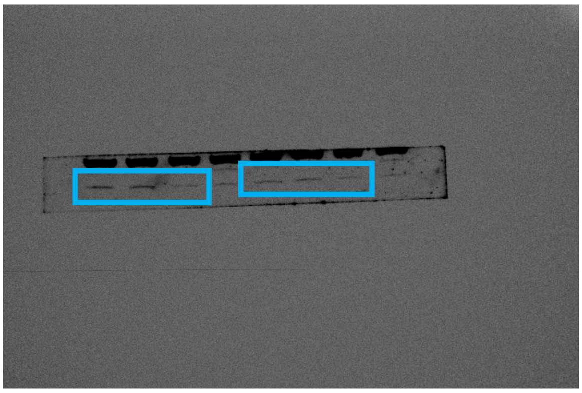

c.PARP

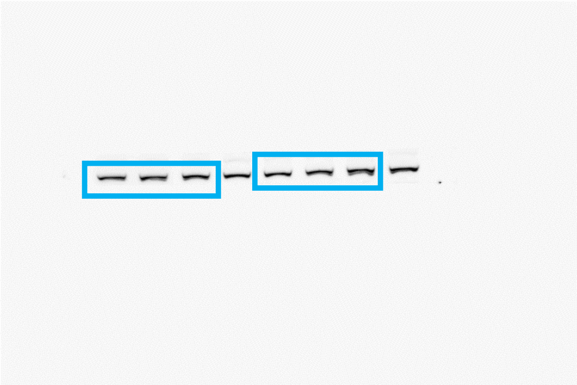

c.Caspase-3

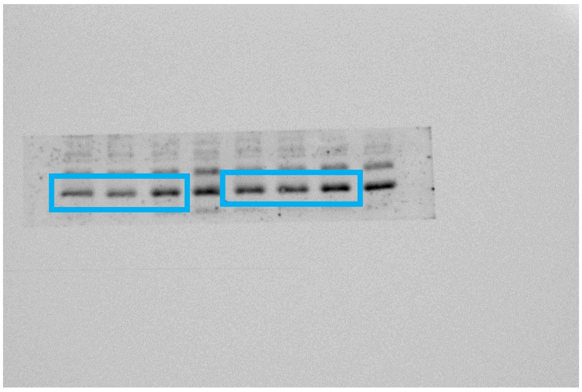

GAPDH

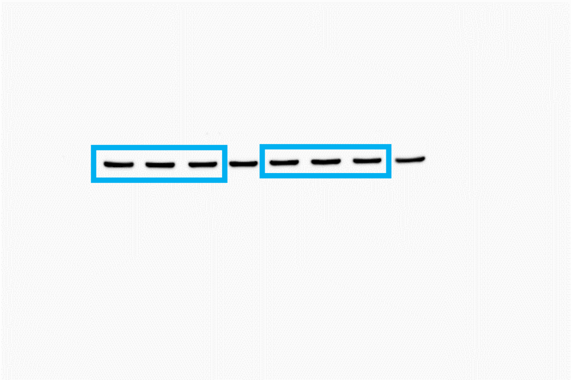

Figure 4D

MYC

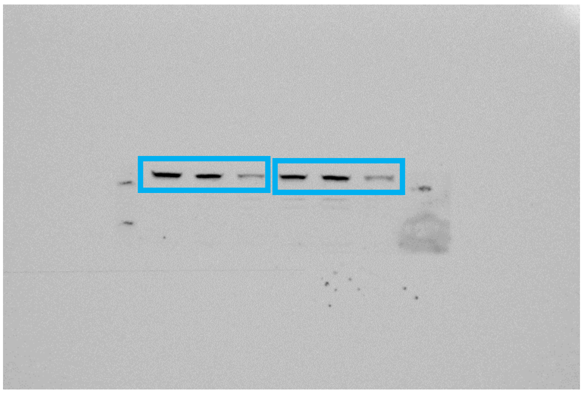

c.PARP

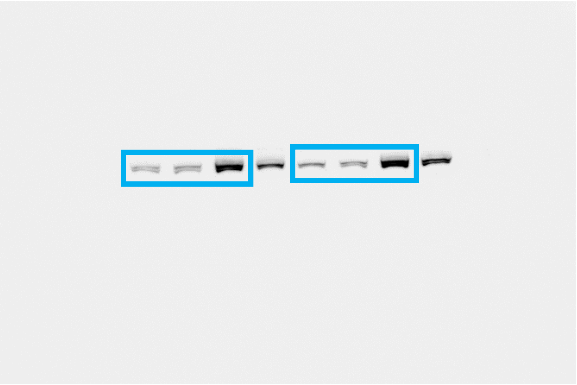

c.Caspase-3

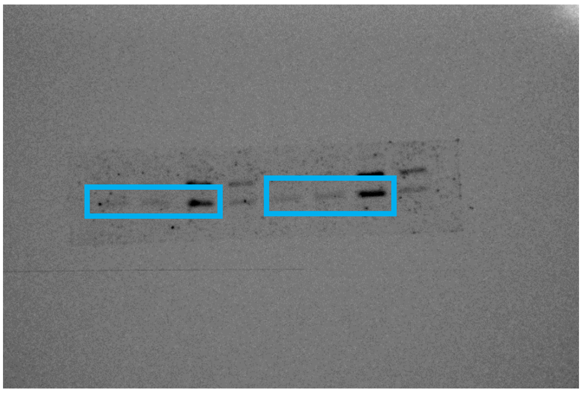

GAPDH

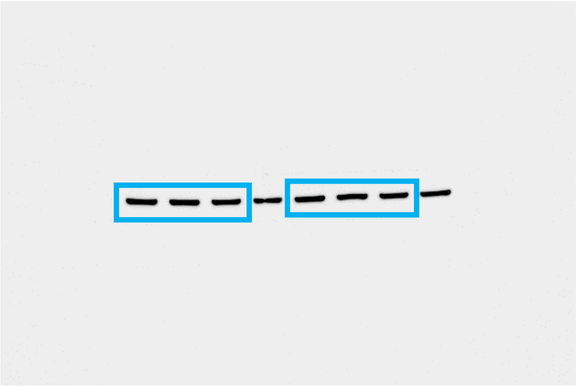

DNA alignment

K562 and K562-IR BCR12-ABL10

Forward sequence

```
Matrix: EDNAFULL
Gap penalty: 2.0
Extend penalty: 2.0
Score: 5222.0
K562 length:1050
K562-IR length:1050
Alignment length: 1054
Identity:    1046/1054 (99.24%)
Similarity:  1046/1054 (99.24%)
Gaps:           8/1054 (0.76%)

1        GGAGTCTCCGGGGGCTCTATGGGTTTCTGAATGTCATCGTCCACTCAGCCACTGGA    60
             ||||||||||||||||||||||||||||||||||||||||||||||||||||||||
1            GGAGTCTCCGGGGGCTCTATGGGTTTCTGAATGTCATCGTCCACTCAGCCACTGGA    59

61       TTTAAGCAGAGTTCAAAAGCCCTTCAGCGGCCAGTAGCATCTGACTTTGAGCCTCAGGGT    120
         ||||||||||||||||||||||||||||||||||||||||||||||||||||||||||||
60       TTTAAGCAGAGTTCAAAAGCCCTTCAGCGGCCAGTAGCATCTGACTTTGAGCCTCAGGGT    119

121      CTGAGTGAAGCCGCTCGTTGGAACTCCAAGGAAAACCTTCTCGCTGGACCCAGTGAAAAT    180
         ||||||||||||||||||||||||||||||||||||||||||||||||||||||||||||
120      CTGAGTGAAGCCGCTCGTTGGAACTCCAAGGAAAACCTTCTCGCTGGACCCAGTGAAAAT    179

181      GACCCCAACCTTTTCGTTGCACTGTATGATTTTGTGGCCAGTGGAGATAACACTCTAAGC    240
         ||||||||||||||||||||||||||||||||||||||||||||||||||||||||||||
180      GACCCCAACCTTTTCGTTGCACTGTATGATTTTGTGGCCAGTGGAGATAACACTCTAAGC    239

241      ATAACTAAAGGTGAAAAGCTCCGGGTCTTAGGCTATAATCACAATGGGGAATGGTGTGAA    300
         ||||||||||||||||||||||||||||||||||||||||||||||||||||||||||||
240      ATAACTAAAGGTGAAAAGCTCCGGGTCTTAGGCTATAATCACAATGGGGAATGGTGTGAA    299

301      GCCCAAACCAAAAATGGCCAAGGCTGGGTCCCAAGCAACTACATCACGCCAGTCAACAGT    360
         ||||||||||||||||||||||||||||||||||||||||||||||||||||||||||||
300      GCCCAAACCAAAAATGGCCAAGGCTGGGTCCCAAGCAACTACATCACGCCAGTCAACAGT    359

361      CTGGAGAAACACTCCTGGTACCATGGGCCTGTGTCCCGCAATGCCGCTGAGTATCTGCTG    420
         ||||||||||||||||||||||||||||||||||||||||||||||||||||||||||||
360      CTGGAGAAACACTCCTGGTACCATGGGCCTGTGTCCCGCAATGCCGCTGAGTATCTGCTG    419

421      AGCAGCGGGATCAATGGCAGCTTCTTGGTGCGTGAGAGTGAGAGCAGTCCTGGCCAGAGG    480
         ||||||||||||||||||||||||||||||||||||||||||||||||||||||||||||
420      AGCAGCGGGATCAATGGCAGCTTCTTGGTGCGTGAGAGTGAGAGCAGTCCTGGCCAGAGG    479

481      TCCATCTCGCTGAGATACGAAGGGAGGGTGTACCATTACAGGATCAACACTGCTTCTGAT    540
         ||||||||||||||||||||||||||||||||||||||||||||||||||||||||||||
480      TCCATCTCGCTGAGATACGAAGGGAGGGTGTACCATTACAGGATCAACACTGCTTCTGAT    539

541      GGCAAGCTCTACGTCTCCTCCGAGAGCCGCTTCAACACCCTGGCCGAGTTGGTTCATCAT    600
         ||||||||||||||||||||||||||||||||||||||||||||||||||||||||||||
540      GGCAAGCTCTACGTCTCCTCCGAGAGCCGCTTCAACACCCTGGCCGAGTTGGTTCATCAT    599

601      CATTCAACGGTGGCCGACGGGCTCATCACCACGCTCCATTATCCAGCCCCAAAGCGCAAC    660
         ||||||||||||||||||||||||||||||||||||||||||||||||||||||||||||
600      CATTCAACGGTGGCCGACGGGCTCATCACCACGCTCCATTATCCAGCCCCAAAGCGCAAC    659

661      AAGCCCACTGTCTATGGTGTGTCCCCCAACTACGACAAGTGGGAGATGGAACGCACGGAC    720
         ||||||||||||||||||||||||||||||||||||||||||||||||||||||||||||
660      AAGCCCACTGTCTATGGTGTGTCCCCCAACTACGACAAGTGGGAGATGGAACGCACGGAC    719

721      ATCACCATGAAGCACAAGCTGGGCGGGGGCCAGTACGGGGAGGTGTACGAGGGCGTGTGG    780
         ||||||||||||||||||||||||||||||||||||||||||||||||||||||||||||
720      ATCACCATGAAGCACAAGCTGGGCGGGGGCCAGTACGGGGAGGTGTACGAGGGCGTGTGG    779

781      AAGAAATACAGCCTGACGGTGGCCGTGAAGACCTTGAAGGAGGACACCATGGAGGTGGAA    840
         ||||||||||||||||||||||||||||||||||||||||||||||||||||||||||||
780      AAGAAATACAGCCTGACGGTGGCCGTGAAGACCTTGAAGGAGGACACCATGGAGGTGGAA    839

841      GAGTTCTTGAAAGAAGCTGCAGTCATGAAAGAGATCAAACACCCTAACCTGGTGCAGCTC    900
         ||||||||||||||||||||||||||||||||||||||||||||||||||||||||||||
840      GAGTTCTTGAAAGAAGCTGCAGTCATGAAAGAGATCAAACACCCTAACCTGGTGCAGCTC    899
```

DNA alignment

K562 and K562-IR BCR12-ABL10

Reverse sequence

```
Matrix: EDNAFULL
Gap penalty: 2.0
Extend penalty: 2.0
Score: 5190.0
Sequence 1 length:1050
Sequence 2 length:1050
Alignment length: 1057
Identity:    1042/1057 (98.58%)
Similarity:  1042/1057 (98.58%)
Gaps:          14/1057 (1.32%)

1                      GCATCTCAGGCACGTCAGTGGTGTCTCTGTGCTCTGCAGCTCT     57
                       |||||||||||||||||||||||||||||||||||||||||||
1                      GCATCTCAGGCACGTCAGTGGTGTCTCTGTGCTCTGCAGCTCT     59

58     CCTGGAGGTCCTCGTCTTGGTGGGCAGCTCTGGGGCCTGCAGCAAGGTACTCACAGCCCC     117
       ||||||||||||||||||||||||||||||||||||||||||||||||||||||||||||
60     CCTGGAGGTCCTCGTCTTGGTGGGCAGCTCTGGGGCCTGCAGCAAGGTACTCACAGCCCC     119

118    ACGGACGCCTTGTTTCCCCAGCTCCTTTTCCACTTCGTCTGAGATACTGGATTCCTGGAA     177
       ||||||||||||||||||||||||||||||||||||||||||||||||||||||||||||
120    ACGGACGCCTTGTTTCCCCAGCTCCTTTTCCACTTCGTCTGAGATACTGGATTCCTGGAA     179

178    CATTGTTTCAAAGGCTTGGTGGATTTCAGCAAAGGAGGGCCGGTCAGAGGGATTCCACTG     237
       ||||||||||||||||||||||||||||||||||||||||||||||||||||||||||||
180    CATTGTTTCAAAGGCTTGGTGGATTTCAGCAAAGGAGGGCCGGTCAGAGGGATTCCACTG     239

238    CCAACATGCTCGCATGAGTTCATAGACCTTCTCTGGGCAGCCTTCTGGGCGCTCCATGCG     297
       ||||||||||||||||||||||||||||||||||||||||||||||||||||||||||||
240    CCAACATGCTCGCATGAGTTCATAGACCTTCTCTGGGCAGCCTTCTGGGCGCTCCATGCG     299

298    GTAGTCCTTCTCTAGCAGCTCATACACCTGGGACAGGTCAATTCCCGGGTAAGGGGACAT     357
       ||||||||||||||||||||||||||||||||||||||||||||||||||||||||||||
300    GTAGTCCTTCTCTAGCAGCTCATACACCTGGGACAGGTCAATTCCCGGGTAAGGGGACAT     359

358    GCCATAGGTAGCAATTTCCCAAAGCAATACTCCAAATGCCCAGACGTCGGACTTGATGGA     417
       ||||||||||||||||||||||||||||||||||||||||||||||||||||||||||||
360    GCCATAGGTAGCAATTTCCCAAAGCAATACTCCAAATGCCCAGACGTCGGACTTGATGGA     419

418    GAACTTGTTGTAGGCCAGGCTCTCGGGTGCAGTCCATTTGATGGGGAACTTGGCTCCAGC     477
       ||||||||||||||||||||||||||||||||||||||||||||||||||||||||||||
420    GAACTTGTTGTAGGCCAGGCTCTCGGGTGCAGTCCATTTGATGGGGAACTTGGCTCCAGC     479

478    ATGGGCTGTGTAGGTGTCCCCTGTCATCAACCTGCTCAGGCCAAAATCAGCTACCTTCAC     537
       ||||||||||||||||||||||||||||||||||||||||||||||||||||||||||||
480    ATGGGCTGTGTAGGTGTCCCCTGTCATCAACCTGCTCAGGCCAAAATCAGCTACCTTCAC     539

538    CAAGTGGTTCTCCCCTACCAGGCAGTTTCGGGCAGCAAGATCTCTGTGGATGAAGTTTTT     597
       ||||||||||||||||||||||||||||||||||||||||||||||||||||||||||||
540    CAAGTGGTTCTCCCCTACCAGGCAGTTTCGGGCAGCAAGATCTCTGTGGATGAAGTTTTT     599

598    CTTCTCCAGGTACTCCATGGCTGACGAGATCTGAGTGGCCATGTACAGCAGCACCACGGC     657
       ||||||||||||||||||||||||||||||||||||||||||||||||||||||||||||
600    CTTCTCCAGGTACTCCATGGCTGACGAGATCTGAGTGGCCATGTACAGCAGCACCACGGC     659

658    GTTCACCTCCTGCCGGTTGCACTCCCTCAGGTAGTCCAGGAGGTTCCCGTAGGTCATGAA     717
       ||||||||||||||||||||||||||||||||||||||||||||||||||||||||||||
660    GTTCACCTCCTGCCGGTTGCACTCCCTCAGGTAGTCCAGGAGGTTCCCGTAGGTCATGAA     719

718    CTCAGTGATGATATAGAACGGGGGCTCCCGGGTGCAGACCCCAAGGAGCTGCACCAGGTT     777
       ||||||||||||||||||||||||||||||||||||||||||||||||||||||||||||
720    CTCAGTGATGATATAGAACGGGGGCTCCCGGGTGCAGACCCCAAGGAGCTGCACCAGGTT     779

778    AGGGTGTTTGATCTCTTTCATGACTGCAGCTTCTTTCAAGAACTCTTCCACCTCCATGGT     837
       ||||||||||||||||||||||||||||||||||||||||||||||||||||||||||||
780    AGGGTGTTTGATCTCTTTCATGACTGCAGCTTCTTTCAAGAACTCTTCCACCTCCATGGT     839

838    GTCCTCCTTCAAGGTCTTCACGGCCACCGTCAGGCTGTATTTCTTCCACACGCCCTCGTA     897
       ||||||||||||||||||||||||||||||||||||||||||||||||||||||||||||
840    GTCCTCCTTCAAGGTCTTCACGGCCACCGTCAGGCTGTATTTCTTCCACACGCCCTCGTA     899
```

Human Cell Line Identification Report

Requester	臺北醫學大學	Receive Date	2024.03.28
Trustee	李嘉華/謝筱巧	Report Date	2024.03.29
Sample Type	□Human Cell Line ■Human gDNA		
Analysis Type	□Comparison between samples ■Comparison with DSMZ database		
Sample Name	K562		
Note			

✧ Workflow：

DNA Extraction → STR PCR Amplify → Capilary Electrophoresis → GeneMapper Analysis → Database Analysis → Report

- DNA Extraction：Use DNeasy Blood & Tissue Kit（QIAGEN, 69504）to perform DNA extraction and purification. The detail operation procedure refer kit’s User Manual.
- Multiplex PCR：Use AmpFLSTR™ Identifiler™ Plus PCR Amplification Kit (ThermoFisher, 4427368)。The Kit can amplified 16 human STR gene loci. The detail operation procedure refer kit’s User Manual.
- Fluorescent Capillary Electrophoresis：The STR PCR product were analyzed with DNA Analyzer 3730XL.
- Data Analysis with GeneMapper v4.0
- STR profiling analysis with **DSMZ** database。

✧ Result：

■ STR loci genotype ：

Locus Name	D5S818	D13S317	D7S820	D16S539	vWA	TH01	AMEL	TPOX	CSF1PO
Allele genotype	11.12	8.8	9.11	11.12	16.16	9.3.9.3	XX	8.9	9.10

■ STR loci genotype comparison result：

◆ Matching Algorithm description：

- The human STR profile database includes data sets of 2455 cell lines from ATCC, DSMZ, JCRB and RIKEN.
- The similarity score is calculated.

$$\frac{2 \cdot A_{shared}}{A_{query} + A_{ref}}$$

- Results highlighted in green are usually authentic as long as the cells do not carry a deficiency in DNA mismatch repair (MMR). Results highlighted in yellow indicate a distant relationship when there is a drift of STR alleles in MMR-negative cell lines. Clearly incorrect cell lines are present when the background is red.

◆ **Conclusion**：

Base on comparison with DSMZ **database, this human cell line was highly identical with**

- K562/Vin（Source：RCB2111）(Similarity 100%）

Similarity	Cell line	Source	Shared	D5S818	D7S820	D13S317	D16S539	vWA	TH01	TPOX	CSF1PO	Amelogenin
		Your query		11, 12	9, 11	8, 8	11, 12	16, 16	9.3, 9.3	8, 9	9, 10	X, X
100 %	K562/Vin	RCB2111	9	11, 12	9, 11	8, 8	11, 12	16, 16	9.3, 9.3	8, 9	9, 10	X, X

✧ Ref1: STR loci genotype result

技術人員 :陳柏翰　　　　實驗室主管: 陳素蓮

GeneMapper 4.0

2024032-cell str

Sample Name	Panel	SQI	SQ
K562	Identifiler_v1		

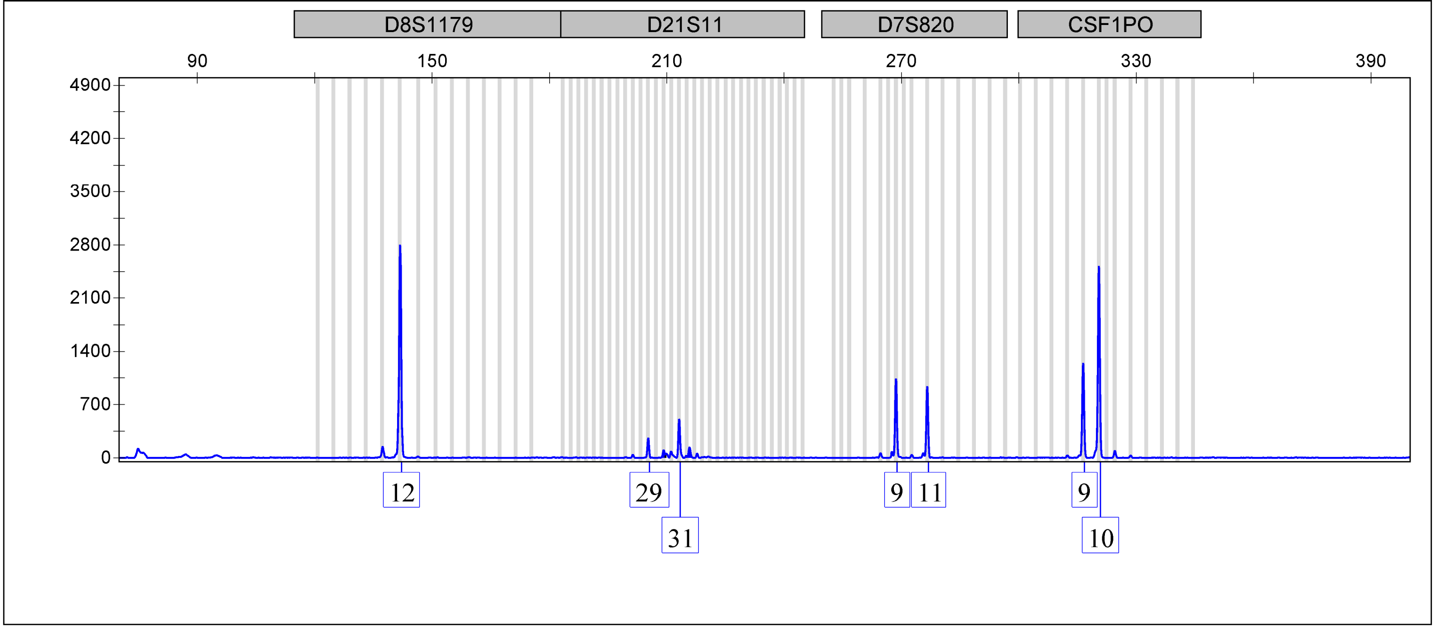

K562	Identifiler_v1		

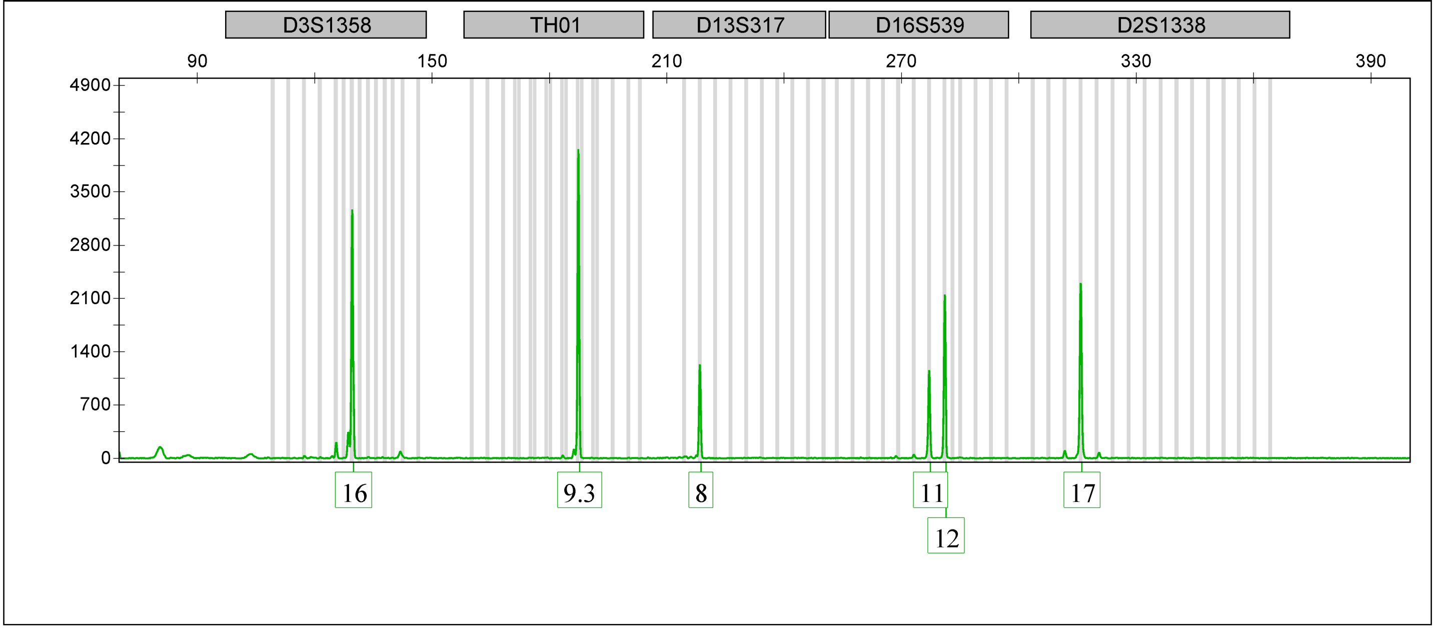

Sample Name	Panel	SQI	SQ
K562	Identifiler v1		

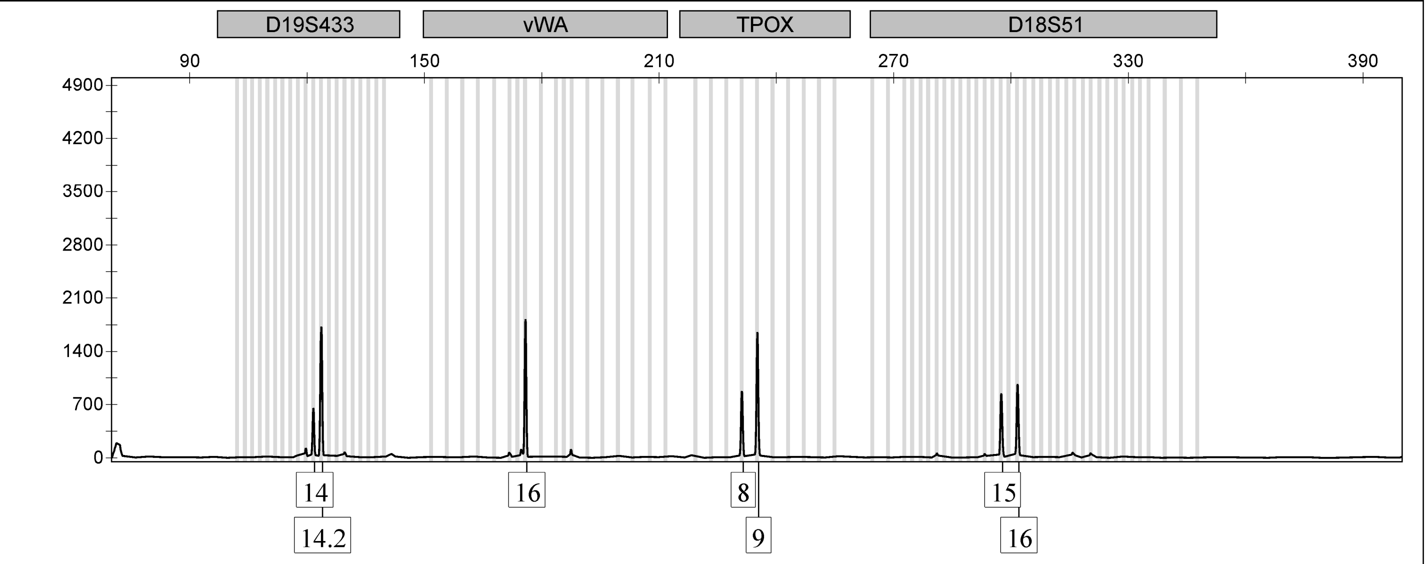

K562	Identifiler v1		

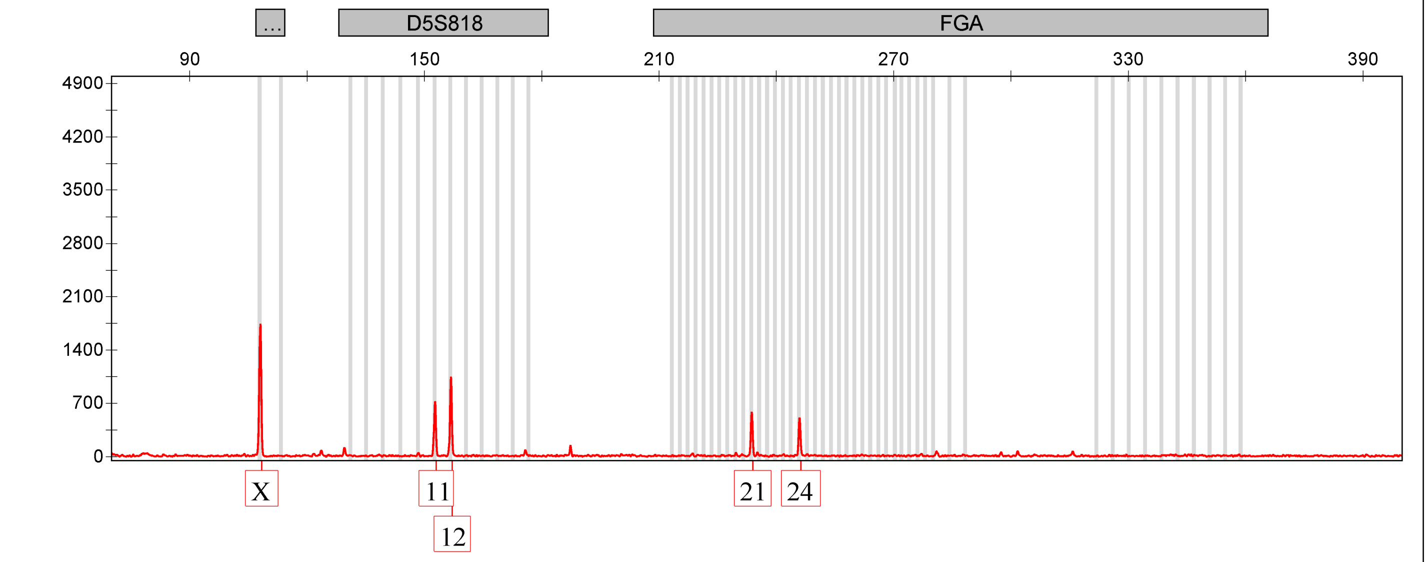

Supplement: Supplementary file 1 [file cells-13-00616-s001.zip › Supplementary Information S2.pdf]
