# Supplementary material for: Unveiling IL6R and MYC as Targeting Biomarkers in Imatinib-Resistant Chronic Myeloid Leukemia through Advanced Non-Invasive Apoptosis Detection Sensor Version 2 Detection
Source: Cells. 2024 Apr 2;13(7):616. doi: 10.3390/cells13070616 (PMC11011921; doi:10.3390/cells13070616)
Supplement: Supplementary file 1 [file cells-13-00616-s001.zip › Supplementary Table S1.pdf]

Supplementary Table S1

Primary Antibody

Antibody	Catalog Number	Company Name (Origin)
**GFP**	GTX113617	GeneTex Inc. (Irvine, CA, USA)
**Firefly luciferase**	GTX125840	GeneTex Inc. (Irvine, CA, USA)
**Firefly luciferase**	GTX125849	Santa Cruz Biotechnology (Santa Cruz, CA, USA)
**GAPDH**	sc-32233	Santa Cruz Biotechnology (Santa Cruz, CA, USA)
**p-ABL**	sc-293130	Santa Cruz Biotechnology (Santa Cruz, CA, USA)
**c-ABL**	sc-23	Santa Cruz Biotechnology (Santa Cruz, CA, USA)
**C-PARP**	#9541	Cell Signaling Technology (Danvers, MA, USA)
**C-CASPASE-3**	#9664	Cell Signaling Technology (Danvers, MA, USA)
**p21**	GTX629543	GeneTex Inc. (Irvine, CA, USA)
**p27**	GTX100446	GeneTex Inc. (Irvine, CA, USA)
**IL6R**	sc-373708	Santa Cruz Biotechnology (Santa Cruz, CA, USA)
**IL6R**	MAB227	R&D System biotechanne
**IL7R**	sc-514445	Santa Cruz Biotechnology (Santa Cruz, CA, USA)
**c-MYC**	#13987	Cell Signaling Technology (Danvers, MA, USA)

Secondary Antibody

goat Anti-Rabbit IgG-HRP	sc-2004	Santa Cruz Biotechnology (Santa Cruz, CA, USA)
**goat Anti-Mouse IgG-HRP**	sc-2005	Santa Cruz Biotechnology (Santa Cruz, CA, USA)
**goat Anti-Rabbit IgG-HRP**	sc-2004	Santa Cruz Biotechnology (Santa Cruz, CA, USA)
