# Supplementary figures and images for: Unveiling IL6R and MYC as Targeting Biomarkers in Imatinib-Resistant Chronic Myeloid Leukemia through Advanced Non-Invasive Apoptosis Detection Sensor Version 2 Detection
Source: Cells. 2024 Apr 2;13(7):616. doi: 10.3390/cells13070616 (PMC11011921; doi:10.3390/cells13070616)

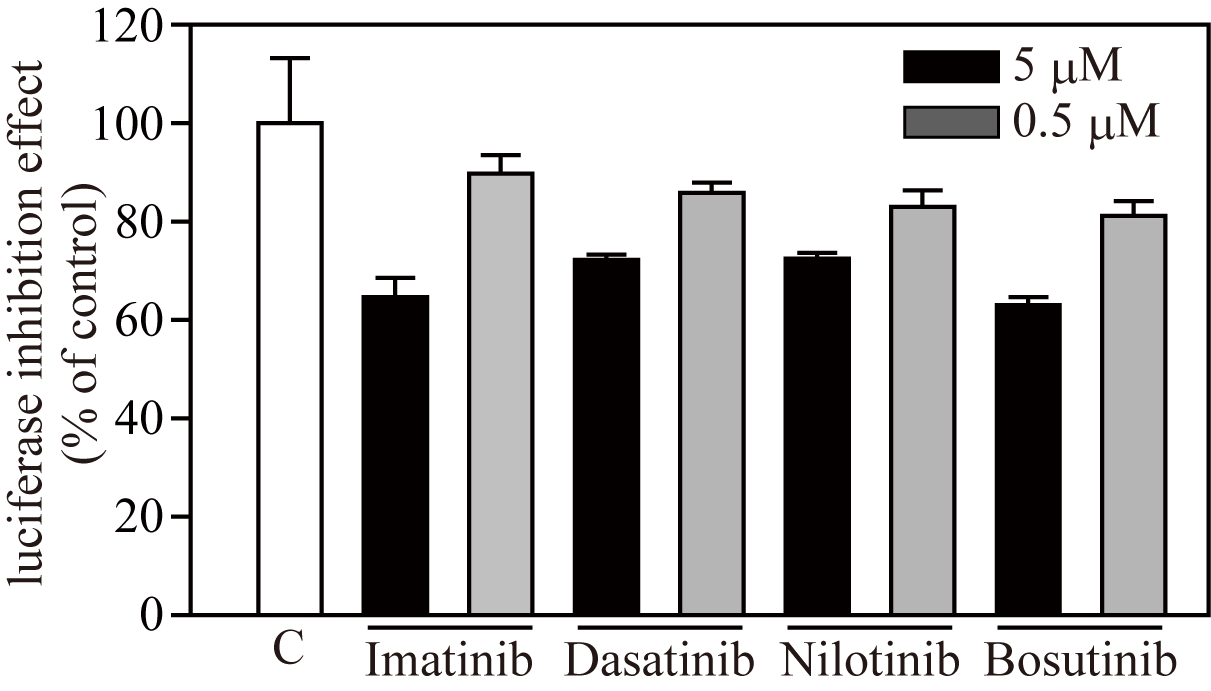

Supplement: Supplementary file 1 [file cells-13-00616-s001.zip › Supplementary Figure S1.tif]

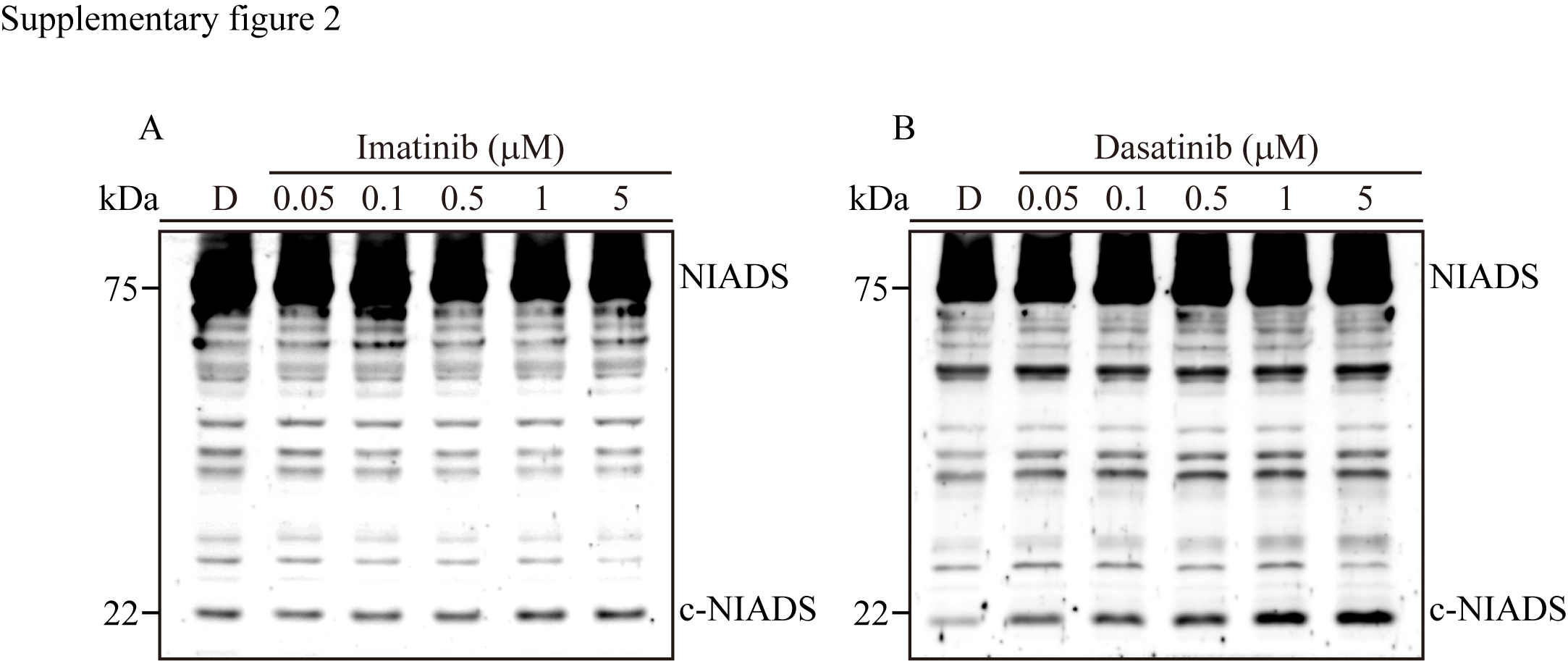

Supplement: Supplementary file 1 [file cells-13-00616-s001.zip › Supplementary Figure S2.tif]

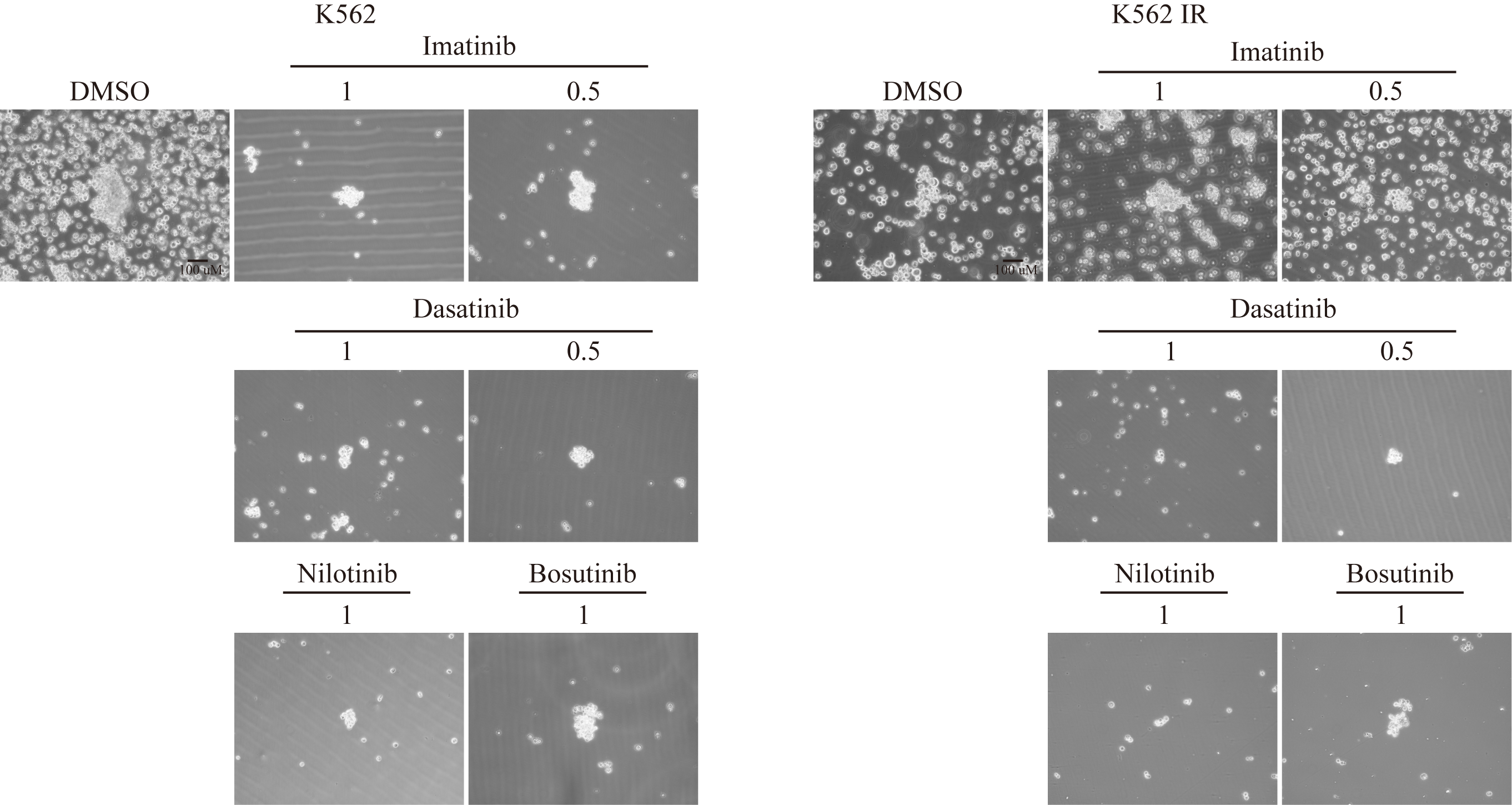

Supplement: Supplementary file 1 [file cells-13-00616-s001.zip › Supplementary Figure S3-20231123.tif]

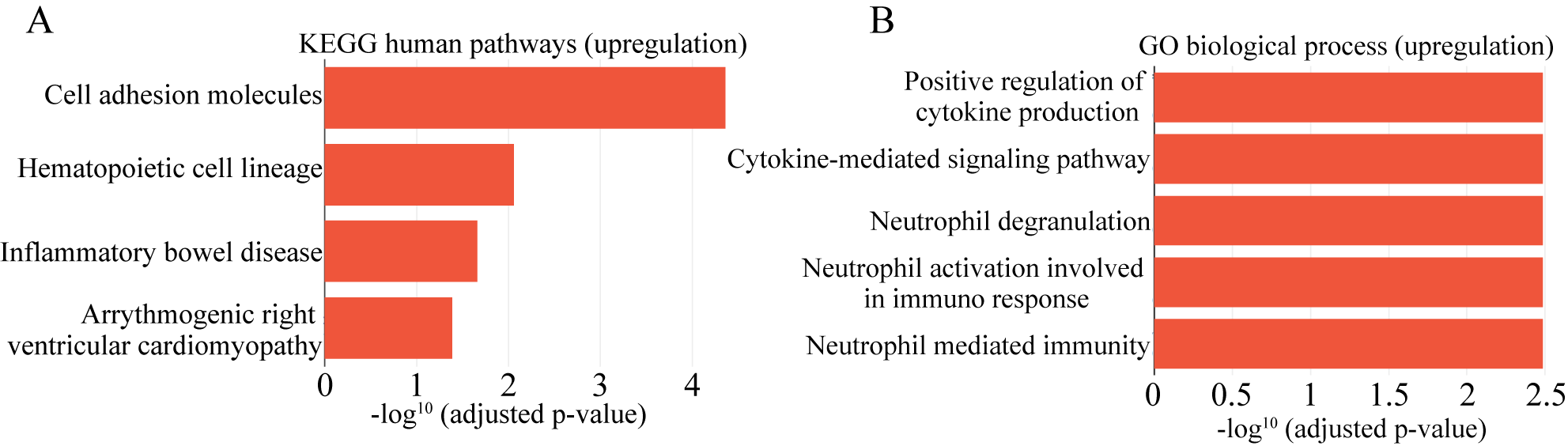

Supplement: Supplementary file 1 [file cells-13-00616-s001.zip › Supplementary Figure S4-20231123.tif]

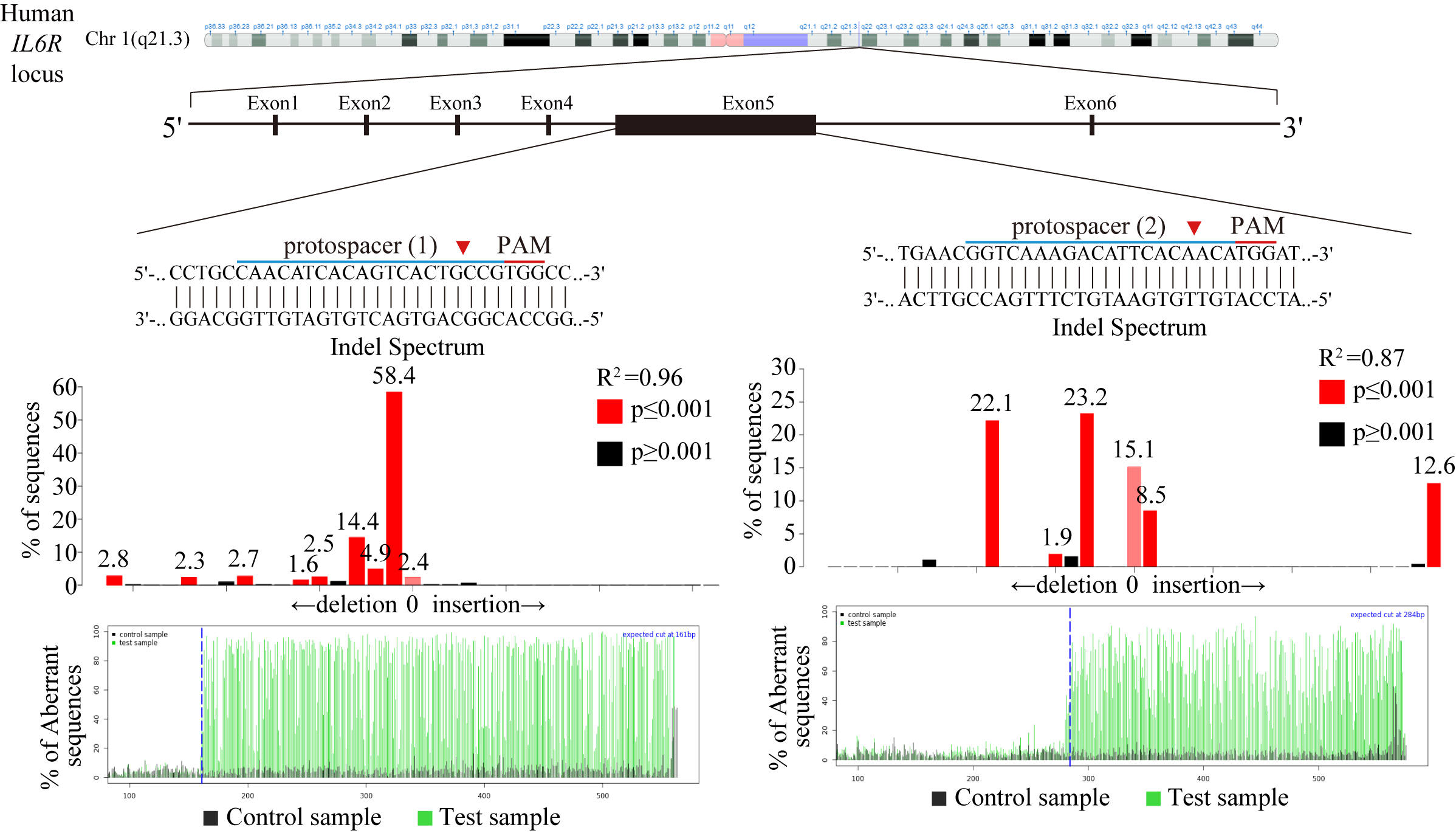

Supplement: Supplementary file 1 [file cells-13-00616-s001.zip › Supplementary Figure S5-20231127.tif]

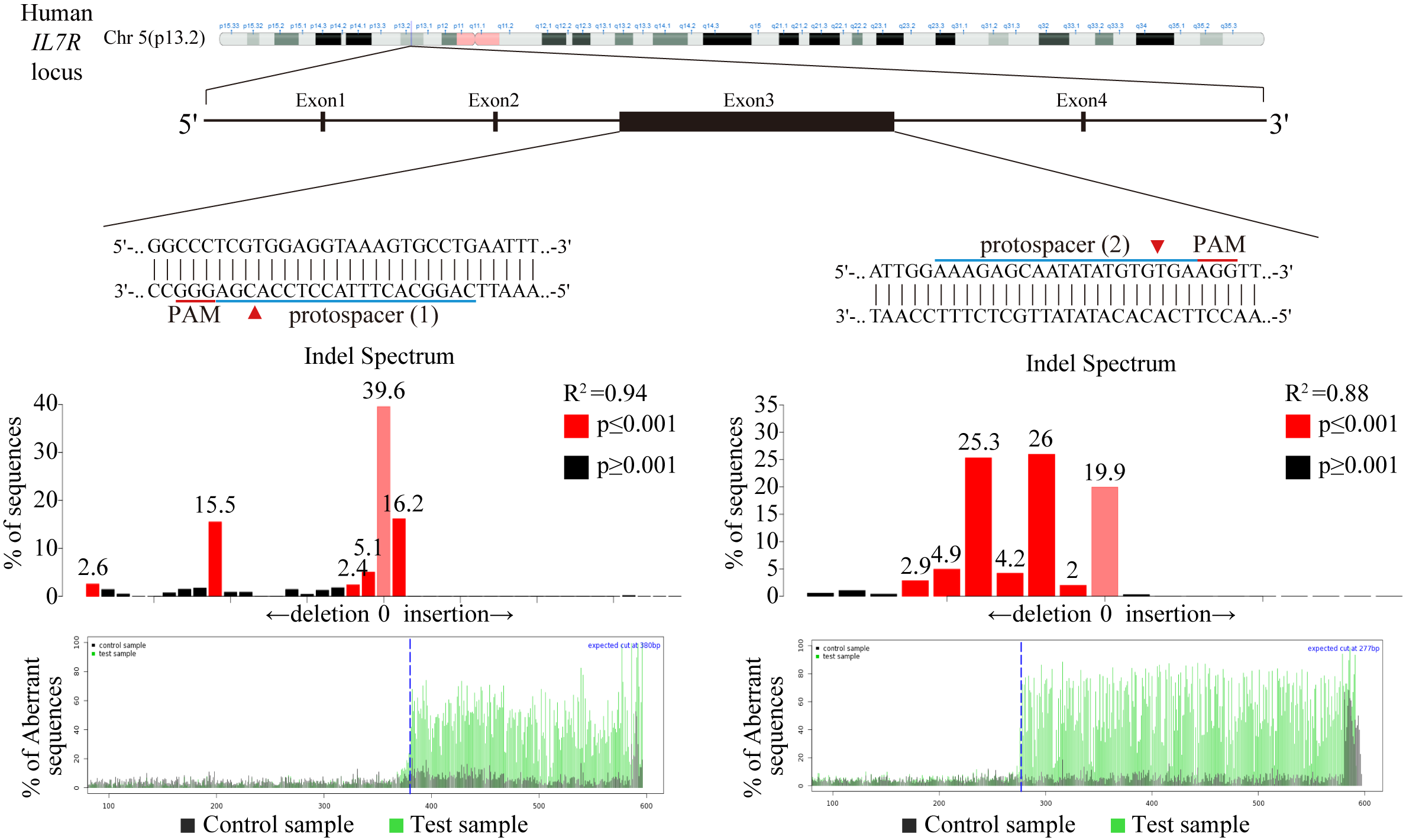

Supplement: Supplementary file 1 [file cells-13-00616-s001.zip › Supplementary Figure S6-20231127.tif]

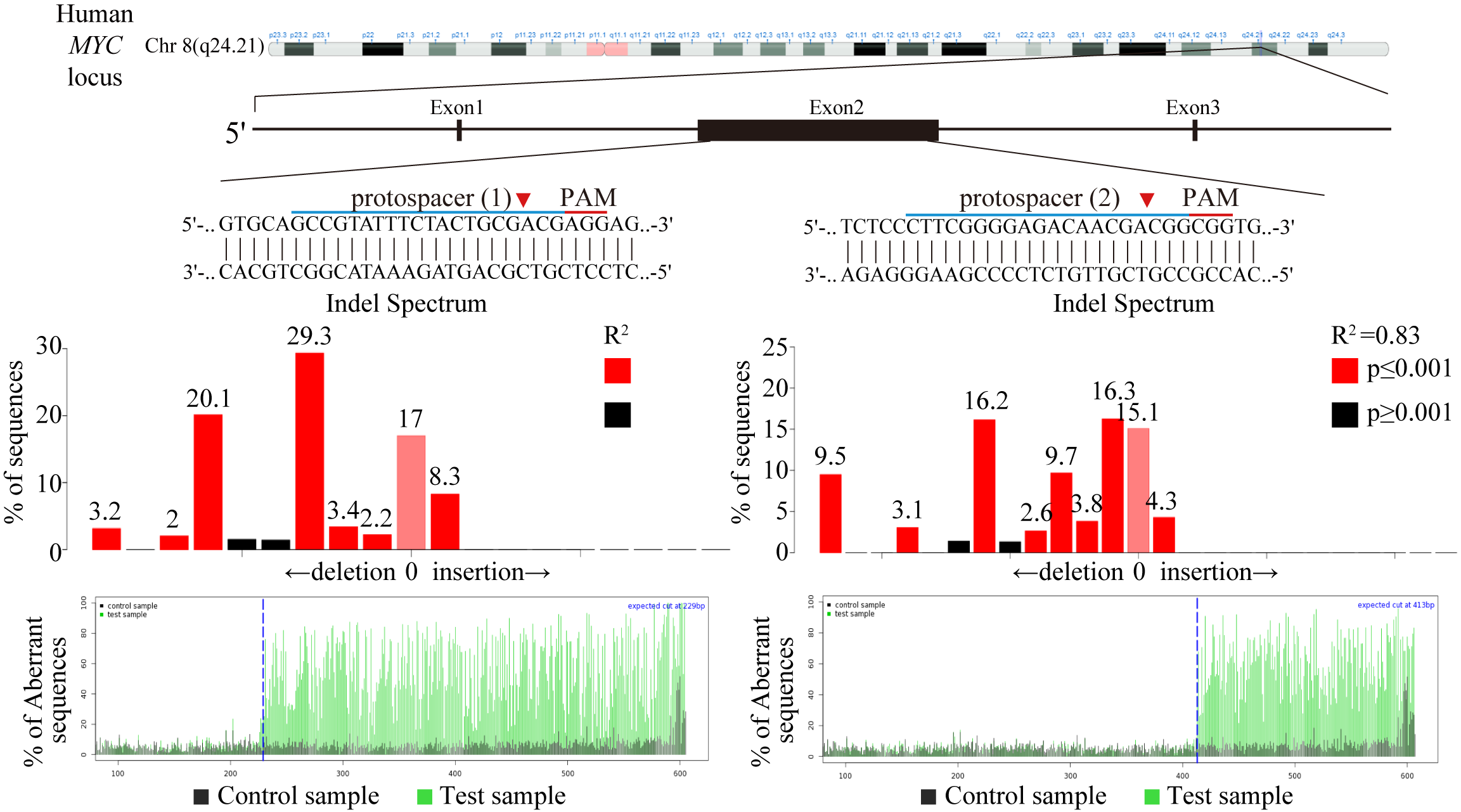

Supplement: Supplementary file 1 [file cells-13-00616-s001.zip › Supplementary Figure S7-20231127.tif]
